# Geographical origin traceability of Cabernet Sauvignon wines based on Infrared fingerprint technology combined with chemometrics

**DOI:** 10.1038/s41598-019-44521-8

**Published:** 2019-06-04

**Authors:** Xiao-Zhen Hu, Si-Qi Liu, Xiao-Hong Li, Chuan-Xian Wang, Xin-Lu Ni, Xia Liu, Yang Wang, Yuan Liu, Chang-Hua Xu

**Affiliations:** 1 College of Food Science & Technology, Shanghai Ocean University, Shanghai, 201306 P.R. China; 2 Shanghai Entry-Exit Inspection and Quarantine Bureau, Shanghai, 200135 P.R. China; 3 First Teaching Hospital of Tianjin University of Traditional Chinese Medicine, Tianjin, 300193 P.R. China; 4 School of Agriculture and Biology, Shanghai Jiaotong University, Shanghai, 200240 China; 5 Department of Pharmacology, Yale University, New Haven, CT 06520 USA; 6 Shanghai Engineering Research Center of Aquatic-Product Processing & Preservation, Shanghai, 201306 P.R. China; 7 Laboratory of Quality and Safety Risk Assessment for Aquatic Products on Storage and Preservation (Shanghai), Ministry of Agriculture, Shanghai, 201306 China; 8 National R&D Branch Center for Freshwater Aquatic Products Processing Technology (Shanghai), Shanghai, 201306 China

**Keywords:** Infrared spectroscopy, Statistics, Infrared spectroscopy, Statistics

## Abstract

Mid-infrared (MIR) and near-infrared (NIR) spectroscopy combined with chemometrics were explored to classify Cabernet Sauvignon wines from different countries (Australia, Chile and China). Commercial wines (n = 540) were scanned in transmission mode using MIR and NIR, and their characteristic fingerprint bands were extracted at 1750-1000 cm^−1^ and 4555-4353 cm^−1^. Through the identification system of Tri-step infrared spectroscopy, the correlation between macroscopic chemical fingerprints and geographical regions was explored more deeply. Furthermore, Principal component analysis (PCA), soft independent modelling of class analogy (SIMCA) and discriminant analysis (DA) based on MIR and NIR spectra were used to visualize or discriminate differences between samples and to realize geographical origin traceability of Cabernet Sauvignon wines. Through “external test set (n = 157)” validation, SIMCA models correctly classified 97%, 97% and 92% of Australian, Chilean and Chinese Cabernet Sauvignon wines, while the DA models correctly classified 86%, 85% and 77%, respectively. Based on unique digital fingerprints of spectroscopy (FT-MIR and FT-NIR) associated with chemometrics, geographical origin traceability was achieved in a more comprehensive, effective and rapid manner. The developed database models based on IR fingerprint spectroscopy with chemometrics could provide scientific basis and reference for geographical origin traceability of Cabernet Sauvignon wines (Australia, Chile and China).

## Introduction

Geographical origin traceability is of great importance in the process of food quality control. At present, the concept of original geographical indication has become one of the important factors affecting consumers’ purchasing preferences in the food and beverage industry^1^. Especially for wine, geographical origin has long been regarded as an inherent criterion in wine identification or classification systems^2,3^. The implementation of comprehensive technologies for origin traceability is urgently needed for consumers, manufacturers, retailers and administrations^1,4,5^. To date, well-established standard means, sensory evaluation, chromatographic and spectrometric methods with expensive instrumentation or time-consuming operation such as gas chromatography (GC), liquid chromatography (LC), mass spectrometry (MS), high performance liquid chromatography (HPLC), elemental and isotopic analysis were applied to discriminate wines on the basis of different geographical origin^1,6–8^.

In contrast, molecular spectroscopy technologies have become attractive technique with a bright prospect in applications of batch detection for wine industry^5,9–11^, due to environmentally friendly characteristics, as well as the potential savings in analysis time and cost. Infrared spectroscopy that could reflect comprehensive information and behaves as a “fingerprint” of the sample, in the MIR spectral region (4000-400 cm^−1^), which is caused by the fundamental stretching, bending, and rotating vibrations of the sample molecules, while NIR spectra (12,800-4000 cm^−1^) results from complex overtones and high-frequency combinations at shorter wavelengths^12–14^. In particular, Tri-step infrared spectroscopy, a comprehensive spectral technique integrating Fourier transform infrared spectroscopy (FT-IR), second derivative infrared spectroscopy (SD-IR) and two-dimensional correlation infrared spectroscopy (2DCOS-IR), has been proved to be an effective technique to reveal main constituents in complicated mixture systems and distinguishing the types and contents of chemical components in highly similar matrices^15–19^. However, few studies focused on the exploration of the key spectral information of origin traceability by recognition mechanism of Tri-step infrared spectroscopy.

Furthermore, multivariate data analysis techniques such as principal component analysis (PCA), cluster analysis (e.g. soft independent modelling of class analogy, SIMCA) and discriminant analysis (DA) can effectively realize the detection of feature patterns or “fingerprint” information related to the geographical origin of samples^5,20^, especially for large-scale sample sets. PCA simplifies the data structure by reducing dimension, which is usually used to detect outliers and to identify patterns in the sample distribution before establishment of classification models^21,22^. While SIMCA and DA are supervised classification methods^5,23^, which have been commonly used in conjunction with MIR and NIR spectroscopy. Previous studies have demonstrated that both the combined application of MIR-SIMCA and the combination of NIR-DA could provide high predictive ability in the analysis of geographical traceability of wines^9,20,24–26^. Therefore, the application of infrared spectroscopy combined with appropriate chemometrics has potential to realize discrimination and traceability of wines with different geographical origins in a more rapid direct and comprehensive manner.

Cabernet Sauvignon (Vitis vinifera L.) is considered as an ancient and traditional red wine grape variety derived its fame from the south west of France^27^. In emerging grape growing regions called New World wine production countries such as Australia, Chile and China, this variety has become an important red cultivar owing to unique flavor characteristics and broad planting area. Particularly, in China, Cabernet Sauvignon has been currently the most famous red grape variety accepted and favored by wine producers and consumers^28^.

Herein, the aim of this study was to explore the macroscopic chemical fingerprints and key spectral information of geographical regions by Tri-step infrared spectroscopy, and further to establish high-throughput classification database models of Cabernet Sauvignon wines with different geographical countries (Australia, Chile, and China) based on unique digital fingerprints of spectroscopy (FT-MIR and FT-NIR) associated with chemometrics.

## Results and Discussion

### Chemical analysis

Supplementary Table S1 showed the one-way variance analysis (ANOVA) for the chemical constituents of Cabernet Sauvignon wines from different countries (Australia, Chile and China). No statistically significant differences between wine samples analyzed with different countries were observed for Alcohol Content (AC), Glucose plus Fructose (G + F), and Total Phenols (TP). However, the differences in pH, Titratable Acidity (TA) and Volatile Acidity (VA) were statistically significant (p < 0.05). By comparing the composition of wines from three countries, Australian Cabernet Sauvignon wines present the highest TA, G + F and TP and the lowest alcohol content, Chilean Cabernet Sauvignon wines present the lowest content of TA, and Chinese Cabernet Sauvignon wines had the highest values of alcohol content, VA and pH, and the lowest content of G + F.

### Tri-step IR spectral analysis

#### IR spectra of three cabernet sauvignon wines

**Figure 1:**
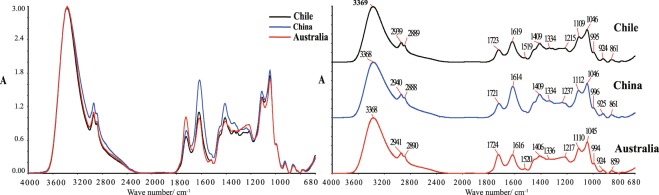
MIR spectra of Cabernet Sauvignon wines from different countries (Chile, China and Australia) in the range of 4000~680 cm^−1^.

According to the one-dimensional FT-MIR spectra (Fig. 1) and the information of corresponding characteristic absorption peaks^29–33^ (see Supplementary Table S2), main constituents of Cabernet Sauvignon wine samples from three countries were considered to be the same. Through the overlapping and separated MIR spectra (Fig. 1), significant differences of multiple characteristic peak intensity and shape among wines from three countries (Chile, China and Australia) were observed, such as peaks at 2940, 2890, 1723, 1618, 1409, 1109 and 1046 cm^−1^, etc. The peak height ratio (1723/1618) of the two main absorption peaks at 1723 and 1618 cm^−1^ were 0.588, 0.475 and 1.091 for Chile, China and Australia (Fig. 1b), which indicated that relative content of the corresponding esters and carboxylic acids were different in wine samples from different countries. Australian Cabernet Sauvignon wines had the largest intensity of *v*(C=O) absorption peak at 1723 cm^−1^, while the peak intensities of stretching vibrational absorption peak of COO− at 1618 and 1409 cm^−1^ were significantly weaker than other two countries. Chinese Cabernet Sauvignon wines had the strongest stretching vibration absorption peaks of C−H bond at 2940 and 2890 cm^−1^, COO− bond at 1618 and 1409 cm^−1^, and C−O bond at 1107 cm^−1^ and 1046 cm^−1^, which indicated that the content of alcohol, glycerol and carboxylic acids were higher than the others. Absorption bands around 1275-1200 cm^−1^ were mainly associated with aromatic compounds and their derivatives, ether-containing compounds. In general, according to the absorption peaks intensities (Fig. 1), Australian Cabernet Sauvignon wines present the highest content of ester and carboxylic acids, and the aromatic substances are abundant. Chinese Cabernet Sauvignon wines had less esters, and the carboxylic acid content was much higher than that of the ester. For Chilean Cabernet Sauvignon wines, the esters content was the lowest, while the alcohol and carboxylic acid content were moderate. It has been indicated that Cabernet Sauvignon wines from different countries present unique flavor personality with different flavor components such as alcohol, ester, acids and carbohydrate.

#### Second derivative IR spectra of three Cabernet Sauvignon wines

**Figure 2:**
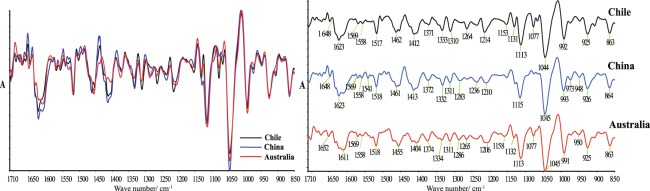
Second derivative spectra of Cabernet Sauvignon wines from different countries (Chile, China and Australia) in the range of 1710~850 cm^−1.^

Generally, the overlapping absorption peaks can be separated, as well as components with low content or weak absorption intensities in the mixture can be more intuitively identified and compared by SD-IR spectra. Characteristic peaks around 1080-1044 cm^−1^ representing the vibration of C-OH bonds of ethanol, glycerol and sugars (G + F) were observed more directly in the SD-IR spectra of Cabernet Sauvignon wines in the range of 1710-850 cm^−1^ (Fig. 2). Moreover, more information about absorbance peaks appeared, such as peak related to aromatic groups at 1265 cm^−1^, *v*(OC=O), *v*(C=C), *v*(C−H_2_), *v*(C−H_3_) absorbance peaks presenting organic acids and aldehyde at 1464-1400 cm^−1^, *v*(C=O) peak associated with free amino acids and peptides at 1650 cm^−1^, and absorption bands of amino acids and their derivatives at 1600-1530 cm^−1^. According to the macroscopic fingerprint differences (peak intensity, position and shape) in SD-IR spectra of three Cabernet Sauvignon wines, it showed that the amino acid and aromatic compounds including their derivatives (phenols) types and contents of Cabernet Sauvignon wines in three countries are significantly different. Compared with Australia and Chile, sugar and phenols contents of the Cabernet Sauvignon wine samples from China were less.

#### 2DCOS-IR spectra of three cabernet Sauvignon wines

To identify differences among the wines from different countries (Chile, China and Australia) more remarkably and convincingly, the synchronous 2DCOS-IR spectra has been applied in the wave number range of 1800~850 cm^−1^. In synchronous 2DCOS-IR spectrum, the peaks show the coincidence of the spectral intensity variations at corresponding variables along the perturbation and can be used to verify differences between samples^15^. The auto-peaks on the diagonal line represented the susceptibility and auto-correlativity of certain absorption bands, which produced changes in spectral intensity by thermal treatment. Positive correlation (red/green area) in 2DCOS-IR spectra indicates that a group of absorption bands change simultaneously (either stronger or weaker), while negative correlation (blue area) is completely the opposite^34–36^.

**Figure 3:**
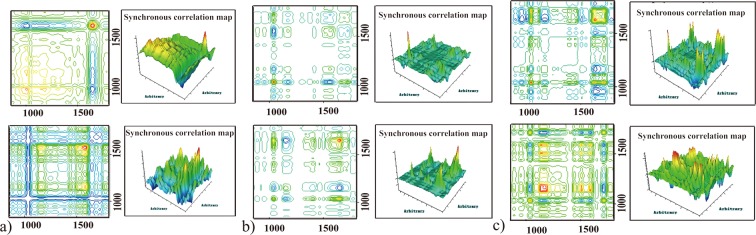
2DCOS-IR synchronous spectra of Cabernet Sauvignon wines from different countries (Chile, China and Australia) in the range of 1800~850 cm^−1.^

Cabernet Sauvignon wines from three countries (Chile, China and Australia) responded differently to thermal perturbation, which made the mean 2DCOS-IR spectra display obvious variation (Fig. 3). The information about positions, relative intensities and correlation of auto-peaks in the temperature ranges of 65–110 °C and 110–120 °C were summarized (see Supplementary Tables S3 and S4). During the thermal perturbation at 65–110 °C, the presence of the strongest auto-peaks at 1648 cm^−1^ and two weak peaks at 1516 cm^−1^ and 1044 cm^−1^ for Chilean Cabernet Sauvignon wines. Australian Cabernet Sauvignon wines had the strongest auto-peak at 1042 cm^−1^ and four weak auto-peaks (1448 cm^−1^, 1408 cm^−1^, 1164 cm^−1^, 965 cm^−1^), whereas Chinese Cabernet Sauvignon wines had one strong auto-peak at 1044 cm^−1^ and four weak auto-peaks (1721 cm^−1^, 1560 cm^−1^, 1512 cm^−1^, 1164 cm^−1^). Furthermore, during the thermal perturbation at 110–120 °C, the presence of the strongest auto-peaks at 1580 cm^−1^ and four weak auto-peaks (1435 cm^−1^, 1250 cm^−1^, 962 cm^−1^, 884 cm^−1^) for Chilean Cabernet Sauvignon wines. Australian Cabernet Sauvignon wines had the strongest auto-peaks at 1160 cm^−1^ and three weak auto-peaks (1408 cm^−1^, 1152 cm^−1^, 1042 cm^−1^), whereas Chinese Cabernet Sauvignon wines had one strong auto-peak at 1650cm^−1^ and three weak auto-peaks (1448 cm^−1^, 962 cm^−1^, 884 cm^−1^). Therefore, Cabernet Sauvignon wines had respective unique fingerprints in the range of 1710-850 cm^−1^ in the synchronous 2DCOS-IR spectra.

Form the all above, the strong automatic peak of Chinese Cabernet Sauvignon wines was mainly the contribution of ethanol and glycerin by the thermal perturbation of 65–110 °C, and the presence of the strongest automatic peak during 110–120 °C was the response of free amino acids and polypeptide components. During the whole process of thermal perturbation, the strongest change in temperature response of Chilean wines was primarily amino acid, followed by aromatic substances (phenols) and glycerol components, while Australian wines was mainly contributed by the ethanol, glycerol and phenolic compounds, followed by esters, carboxylic acids and aromatic amino acids.

According to the response intensity of different components to the thermal perturbation in the 2DCOS-IR spectra, geographical differences between the Cabernet Sauvignon wines could be directly judged. Therefore, Chillan, Chinese and Australian wines could be identified and distinguished more clearly and completely based upon the MIR macro-fingerprint characteristics.

#### PCA analysis

**Figure 4:**
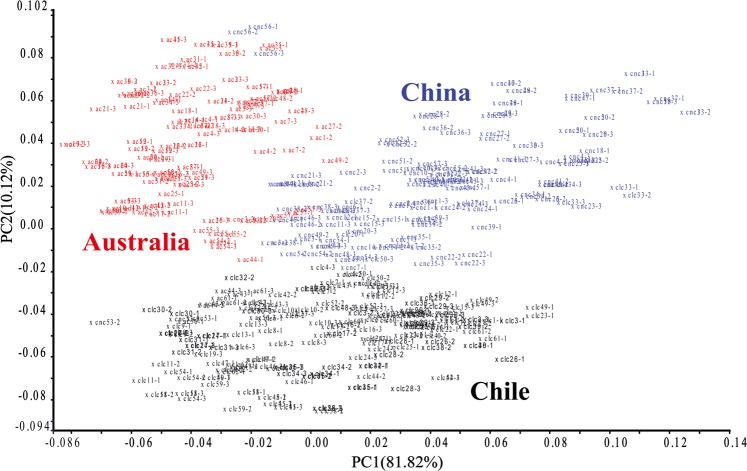
Score plot of the first two PCs for Cabernet Sauvignon wines from different countries (CLC: Chile; CNC: China; AC: Australia) based on FT-MIR spectra.

Combining the spectral characteristics extracted from Tri-step IR analysis, the pre-processed MIR spectra (Standard Normal Variate, SNV and De-trending, 1710-850 cm^−1^) of the Cabernet Sauvignon wine samples were analyzed by PCA (Fig. 4), which could visualize systematic differences between samples from the three countries. The first two PCs displayed almost 92% of total variance in the selected wine samples from three countries. Three clusters representing all wine samples (Chile, China, and Australia) were observed, however, some samples mainly from China and Australia did overlap. To investigate the potential relationship between characteristics of origin and specific chemical composition, the PCA eigenvectors and the spectral characteristics (see Supplementary Fig. S1) were analyzed. PC1 explained 81.82% of the total variance, and the highest loadings were located in the absorption band around 1080-1045 cm^−1^, which represented the vibration of C-OH band. Additional absorption bonds were observed at 1650 cm^−1^ and 864 cm^−1^, associated with C=O and −CH bands, respectively. In PC2 (10.12%), the highest eigenvectors were observed at around 1650-1620 cm^−1^ and 1100-980 cm^−1^. Therefore, the PCA analysis for Australian, Chillan and Chinese Cabernet Sauvignon wines were performed based on spectral fingerprint information of MIR. It further demonstrated that alcohols, carbohydrates (glucose and fructose), organic acid and phenolic compounds contributed the strongest differences among the Cabernet Sauvignon wines from different countries (Australia, Chile and China).

#### Cluster analysis

In order to realize cluster analysis of Cabernet Sauvignon wine samples from three countries, SIMCA was performed on the spectral characteristics of wine samples extracted by PCA. 540 parallel samples were objectively classified, and 157 samples (54 samples for Australia, 55 for Chile, 48 for China) were randomly selected as external validation set. The parameters of SIMCA model were summarized (Supplementary Table S5). The between-class distance between the Cabernet Sauvignon wine samples from three countries was >1 (Supplementary Table S6), which indicated that the degree of separation between various categories was relatively large, and the difference of categories was obvious in the SIMCA model. In addition, the reliability of classification model has been verified by recognition and rejection rate.

According to the classification result of SIMCA model (Table 1, Supplementary Table S9), the recognition rate of calibration set and the rejection rate of validation set in samples with three countries were 100%, which demonstrated that the sensitivity of the calibration set and the specificity of the validation set in SIMCA model were accurate. Nevertheless, the recognition rate of the verification set in Chinese samples was only 73%. Correct classification rates for Australian, Chilean and Chinese wines were 97%, 97%, and 92%, respectively. It has been showed that Cabernet Sauvignon wines from three countries were classified effectively by MIR coupled with PCA-based SIMCA.

**Table 1:** Classification performance report of Calibration (C) and Validation (V) for discriminating Cabernet Sauvignon wines from different countries based on MIR.

Cabernet Sauvignon wines	% Recognition rate (C)	% Rejection rate(C)	% Recognition rate (V)	% Rejection rate (V)
Australia	100 (123/123)	99 (248/251)	91 (49/54)	100 (103/103)
Chile	100 (131/131)	97 (236/243)	89 (49/55)	100 (102/102)
China	100 (120/120)	100 (254/254)	73 (35/48)	100 (109/109)

#### Discriminant analysis (DA)

**Figure 5:**
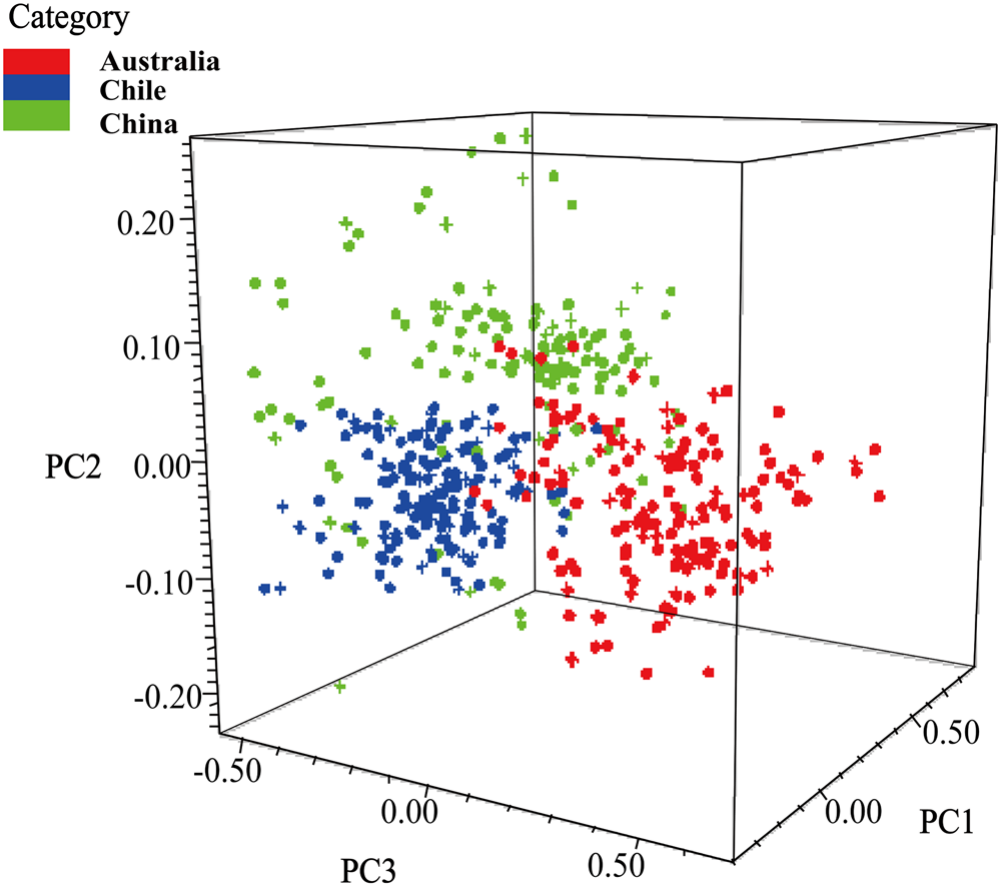
Three-dimensional PCA plot for Cabernet Sauvignon wines from different countries (Australia (AC), Chile (CLC) and China (CNC)) based on NIR spectra.

Supplementary Tablesnon wine samples (406 samples for the calibration set and 134 samples for the validation set) were analyzed using the Mahalanobis distance discriminant analysis (DA). According to the NIR spectral information and optimization analysis (see Supplementary Tables S7^2,37–39^ and S8), the pre-processed NIR spectra (SNV, 4555-4353 cm^−1^) of the Cabernet Sauvignon wine samples were selected for modeling (Supplementary Fig. S2). Through the projection and distribution of the wine samples in the three-dimensional feature space (Fig. 5), the samples of three countries were distinguished. Furthermore, the clustering trend based on FT-NIR is basically consistent with the results of FT-MIR.

**Figure 6:**
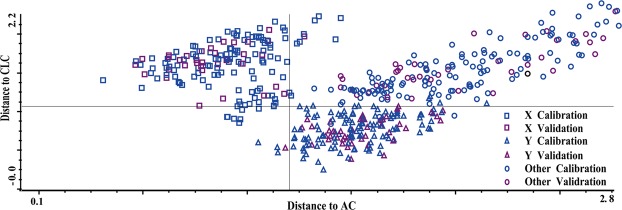
Classification of three Cabernet Sauvignon wines from different countries using Mahalanobis distance discriminant analysis method (□)AC; (△)CLC; (○) CNC.

Cabernet Sauvignon wine samples from three geographical countries were classified based on Mahalanobis distance discrimination (Fig. 6). Correct classification rates for Australian, Chilean and Chinese Cabernet Sauvignon wines were 86%, 85% and 77% (Table 2), respectively. Consistent with SIMCA model based on FT-MIR, prediction effects of Australian and Chilean wines were better than Chinese wines. Furthermore, the accuracy and sensitivity of the SIMCA model based on FT-MIR were better than that of the DA model using FT-NIR.

**Table 2:** Classification performance report of Calibration (C) and Validation (V) for discriminating Cabernet Sauvignon wines from different countries based on NIR.

Cabernet Sauvignon wines	% Recognition rate (C)	% Rejection rate (C)	% Recognition rate(V)	% Rejection rate (V)
Australia	87 (117/135)	100 (258/258)	84 (37/44)	100 (90/90)
Chile	85 (111/130)	91 (240/263)	84 (46/55)	89 (70/79)
China	78 (100/128)	91 (241/265)	71 (25/35)	88 (87/99)

## Conclusion

In this study, we have attempt to establish high-throughput classification models of Cabernet Sauvignon wines with three geographical countries based on unique digital fingerprints of spectroscopy (FT-MIR and FT-NIR) associated with chemometrics.

Through the identification system of Tri-step mid infrared spectroscopy technology, with chemical analysis results of alcohol, pH, total acid, volatile acid, total phenol, glucose and fructose as reference, the macroscopic characteristic fingerprint bands of different countries were extracted at 1750-1000 cm^−1^. As the increasing resolution of Tri-step infrared spectroscopy, apparent differences in the Cabernet Sauvignon wine samples have been fully visualized due to the fingerprint information (positions and relative intensities of characteristic peaks). Three Cabernet Sauvignon wines from different countries were successfully discriminated and classified in a rapid and holistic manner. Moreover, 540 Cabernet Sauvignon wine samples have been objectively used by chemometrics (PCA, SIMCA and DA) based on MIR and NIR macro-fingerprints to realize rapid traceability analysis of unknown Cabernet Sauvignon wine samples from Australia, Chile and China. These results suggested that the prediction effects of Australian and Chilean wines were better than that of Chinese wines, and the classification effect of SIMCA model based on FT-MIR was more precise than DA model based on FT-NIR. For the detection of Chinese wines, it is necessary to improve the accuracy of the classification by establishing robust models with more samples.

Conclusively, it has been demonstrated that the developed database models based on FT-MIR and FT-NIR coupled with chemometrics (PCA, SIMCA and DA) could be applied as a reference for geographical origin traceability of Cabernet Sauvignon wines (Australia, Chile and China) in a more comprehensive, effective and rapid manner.

## Experimental

### Samples and reference data

Commercial Cabernet Sauvignon wine samples from Australia (South Australia, Western Australia, Victoria and New South Wales, respectively), Chile (Central Valley) and China (Hebei, Shandong and Ningxia, respectively) were collected from commercial wineries and Shanghai Entry-Exit Inspection and Quarantine Bureau. A total of 540 wine samples includig Australian (61 labels × 3 bottles), Chilean (62 labels × 3 bottles) wines and Chinese (57 labels × 3 bottles) wines were used in this study. All samples were from 2010 to 2016 vintages, produced in the main wineries of the geographical zones: Penfolds, Capel Vale, McWilliam’s, Vina Ventisquero, Concha y Toro, Montes, Baron Philippe de Rothschild, ChangYu, Greatwall Manor, Chateau Bacchus, Imperial Horse and Dynasty. Reference data for alcohol content (AC), glucose and fructose (G + F), pH, titratable acidity (TA), volatile acidity (VA) and total phenols (TP) were obtained using standard methods (GB 15037-2006)^31,40^.

### Instrument

FT-IR spectrometer (Spotlight 400, PerkinElmer, UK) equipped with a deuterated triglycine sulfate (DTGS) detector and Universal ATR sampling accessory. Thermo Scientific Nicolet iS5 FT-IR spectrometer equipped with ATR temperature controller which performed the thermal perturbation was used to obtain the two-dimensional correlation spectra. The IR spectra were recorded from 4000 to 400 cm^−1^ and 12,800-4000 cm^−1^. Spectra were recorded with 32 scans and 0.5 cm/s^−1^ of OPD speed.

Other equipments include ultrapure water machine (Milli-Q Reference, Elix Reference), Seven Excellence S400-B multifunction tester (METTLER TOLEDO, Switzerland), LC-20A high performance liquid chromatograph (SHIMADZU, Japan), T70 full-automatic potentiometric titrometer (0.01 pH) (METTLER TOLED, Switzerland), Evolution™ 300 UV-Visible spectrometer (Thermo Fisher Scientific, USA), DMA4500 digital densitometer (Anton-Paar, Austria) and rotary evaporator (IK ARV10 basic, HB10 digital, German) and Freeze Dryer BTP-3XLOVX (Virtis, American).

### Procedure

#### Spectroscopic measurements of FT-MIR

3 mL of each wine sample was taken from freshly opened bottles and distilled at 40 °C for about 8 min by rotating evaporator until the resulting wine sample was essentially alcohol-free. Then the resulting sample was freeze-dried for 24 h, each sample (1~2 mg) was mixed with KBr (100 mg) into powder and finally pressed into tablets. FT-IR spectra of samples were scanned at room temperature by PerkinElmer FT-IR spectrometer in transmission mode. Each spectrum was recorded as the average of 32 scans with 4 cm^−1^ resolution in the wavenumber range of 4000-400 cm^−1^. The SD-IR spectra was obtained by Savitzky-Golay polynomial fitting (13-point smoothing) with PerkinElmer Spectrum software (Version 10.4.3).

In order to obtain 2DCOS-IR spectra representing the overall difference of selected samples, each wine sample from three countries was placed in ATR accessory connected with the temperature controller and recorded in two variable temperature-gradients: from 65 to 110 °C with an increasing rate at 2 °C/min at an interval of 10 °C, and from 110 to 120 °C with an increasing rate at 2 °C/min at an interval of 2 °C. After removing the abnormal sample information, a series of mean dynamic spectra of three countries in variable temperature-gradients were processed using 2DCOS-IR correlation analysis software (Thermo Scientific, Nicolet iN10 SpectraCorr). Then, the mean 2DCOS-IR spectra of three countries were obtained.

#### Spectroscopic measurements of FT-NIR

Wine Samples were scanned in transmission mode using near-IR fiber-optic probe accessory of Nicolet iS50 (Thermo Fisher Scientific, America) equipped with an indium-gallium-arsenide (InGaAs) detector. Each spectrum was recorded as the average of 32 scans with 4 cm^−1^ resolution in the wavenumber range of 12,800-4000 cm^−1^. Spectra of all samples were collected at room temperature with reference background spectrum recorded using air. All the raw FT-NIR data were processed with Omnic spectrum software (Version 9.2.106).

#### Multivariate data analysis

A total of 540 dry wine samples (183 samples for Australia, 186 for Chile, 171 for China) were used to establish models of geographical origin traceability by PCA, SIMCA and DA. PCA was conducted based on FT-MIR spectra (1710-850 cm^−1^) by Spectrum Quant+ v 4.6.0.0176 (Perkin Elmer Inc.). SIMCA models (FT-MIR) based on PCA were performed by Assure ID v 4.1.0.0195 (PerkinElmer Inc.). And Mahalanobis distance discriminant models (FT-NIR) based on PCA were developed by TQ Analyst v 9.7.179 (Thermo Fisher Scientific Inc.). Statistical analysis of chemical components was analyzed by SPSS Statistics (Version 23.0) software.

## Supplementary information

Supplementary Information

## Data Availability

The dataset generated or analyzed during the current study are available from corresponding author upon reasonable request.
