# Supplementary material for: Geographical origin traceability of Cabernet Sauvignon wines based on Infrared fingerprint technology combined with chemometrics
Source: Sci Rep. 2019 Jun 4;9:8256. doi: 10.1038/s41598-019-44521-8 (PMC6547656; doi:10.1038/s41598-019-44521-8)
Supplement: Supplementary file 1 — Supplementary Information [file 41598_2019_44521_MOESM1_ESM.pdf]

Geographical origin traceability of Cabernet Sauvignon wines based on Infrared fingerprint technology combined with chemometrics

Xiaozhen Hu[a] [†], Siqi Liu[a,b][†], Xiaohong Li[b], Chuanxian Wang[b], Xinlu Ni[b], Xia Liu[b], Yue Zhang[c], Yuan Liu[d*], Chang-Hua Xu[a, e,f,g,h *]

[a] *College of Food Science & Technology, Shanghai Ocean University, Shanghai 201306, PR China*

[b] *Shanghai Entry-Exit Inspection and Quarantine Bureau, Shanghai, 200135, PR China*

c First Teaching Hospital of Tianjin University of Traditional Chinese Medicine, Tianjin 300193, RP China

[d] *School of Agriculture and Biology, Shanghai Jiaotong University, Shanghai, 200240, China*

[e] *Department of Pharmacology, Yale University, New Haven, CT, 06520, US*

[f] *Shanghai Engineering Research Center of Aquatic-Product Processing & Preservation, Shanghai 201306, PR China*

[g] *Laboratory of Quality and Safety Risk Assessment for Aquatic Products on Storage and Preservation (Shanghai), Ministry of Agriculture, Shanghai 201306, China*

[h] *National R&D Branch Center for Freshwater Aquatic Products Processing Technology (Shanghai), Shanghai 201306, China*

[†] These authors contributed equally to this work.

[*] Corresponding authors: Chang-Hua Xu, Yuan Liu

Tel: 021-61900380

E-mail: chxu@shou.edu.cn, chang-hua.xu@yale.edu , y_liu@sjtu.edu.cn

Supplementary Figures

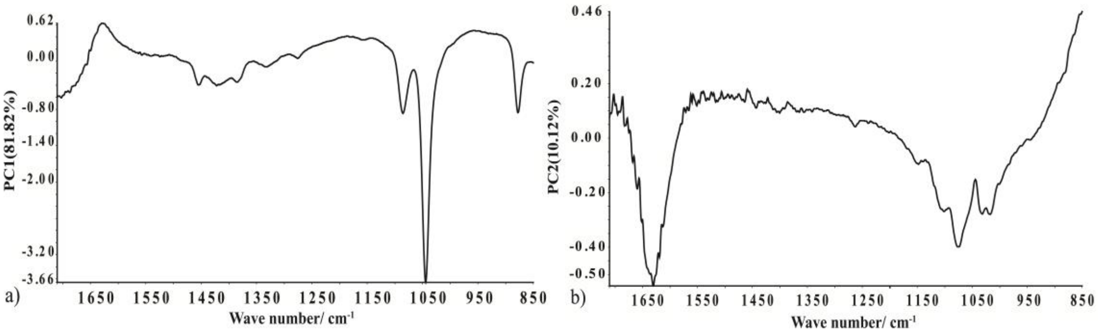

Fig. **1**. Eigenvectors of PC1 (a) and PC2 (b) of Cabernet Sauvignon wines analyzed by MIR.

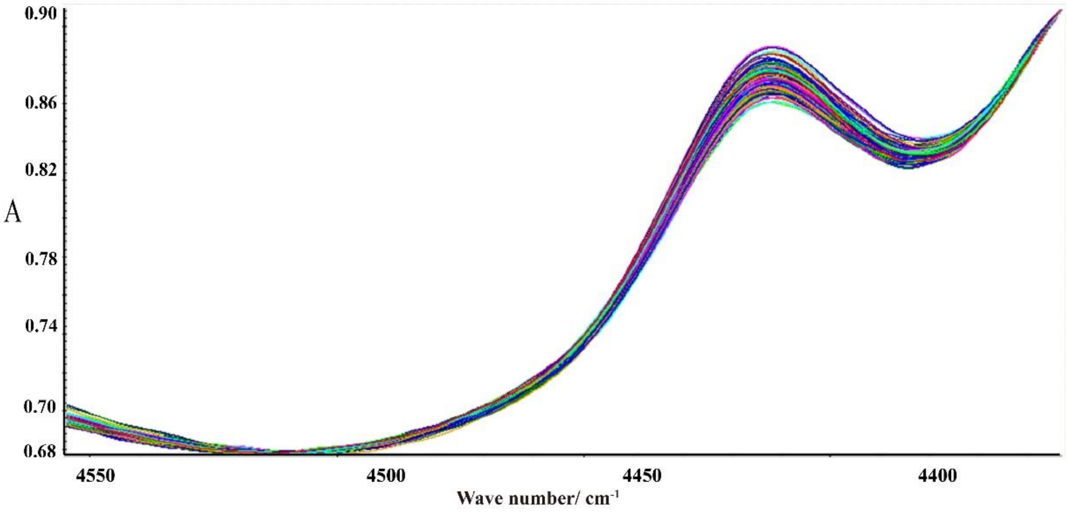

Fig. 2. NIR spectra (4555-4353 cm^{-1}) of Cabernet Sauvignon wines from different countries.

Supplementary Tables

Table 1 Chemical composition of dry red wines from different countries (Chile, China and Australia)

Parameter	Reference method	Chile	China	Australia
Alcohol content (%)	Densimeter method	13.63±0.19[a]	13.99±0.31[a]	13.45±0.43[a]
**pH**	HPLC	**3.49±0.33[a]**	**3.82±0.38[b]**	**3.42±0.28[a]**
G+F (g/L)	Autotitration	1.21±0.30[a]	0.49±0.08[a]	1.52±0.69[a]
**TA (g/L)**	Automatic potentiometric titration	**5.22±0.88[a]**	**5.49±0.15[a]**	**6.28±0.10[b]**
**VA (g/L)**	Automatic potentiometric titration	**0.51±0.03[a]**	**0.62±0.04[b]**	**0.52±0.04[a]**
TP (mg/L)	Folin-Ciocalteu colorimetry	2624.62±135.89[a]	2790.83±184.28[a]	3248.26±162.44[a]

SEL: standard error of laboratory method; G+F: glucose plus fructose; TA: titratable acidity; VA: volatile acidity; TP: total phenols; Values in the table denote the mean ± standard error; [a,b]Levels not connected by same letter are significantly different, $p < 0.05$.

Table 2 The preliminary assignment of main characteristic absorption peaks of FT-MIR spectra of Cabernet Sauvignon wine.

Peak Position（cm^{-1}）	Base Group and Vibration Mode	Main attribution
3300-3500	v (O-H)	Water, Ethanol, Phenols
2985	v (C-H)	Ethanol
2940, 2890	v (CH_3), v (CH_2)	Ethanol, Polyol (glycerol), Fatty acid, Catechin
1750-1710	v (C=O)	Ester
1723	v (C=O)	Esters, Organic carboxylic acids, Aldehydes
1650	v (C=O)	Free amino acid, Polypeptide
1618	$V_{as,s}$ (COO)	Carboxylic acid, Carboxylate, Ester
1608	v (C=C) of Benzene ring	Aromatic amino acids, Phenols (such as gallic acid, catechins)
1600-1530	v_{as} (COO), v (C-N), v (C=C) of Benzene ring	Amino acids and their derivatives
1518	v (O-H), v (C=C), Benzene ring skeleton vibration	Aromatic compounds
1456	δ(CH_3), δ_s(CH_2)	
1454-1419	v (CO=O), v (C=C), v (CH_3), v (CH_2)	Organic carboxylic acid, Aldehyde
1409	v (COO)	Carboxylic acid, Carboxylate, Ester
1375±10	$\delta_{in\text{-}plane}$ (CH_3)	
1333	δ(C-H), v (CH_2)	
1285-1278	$\delta_{in\text{-}plane}$ (O-H)	Aromatic compounds

1275-1200	v (O-H), $\delta_{in\text{-}plane}$(C-H)<br>v (C-O), v (C-X), v (C-C)	Aromatic compounds and their derivatives (phenols), ether-containing compounds
1159, 1131	v (C-O)	Phenols
1110-1107	v(C-O)	Glycerol, Lactic acid, β-amino acids
1077	v (C-O), v (O-H)	Glycerol, Carbohydrate
1045	v (C-O)	Ethanol, Glycerol
1030	v (C-O)	Glucose
994, 924, 860	$\delta_{out\text{-}plane}$ (C-H)	Glycerol, Mono-substituted olefins
970, 950	$\delta_{out\text{-}plane}$ (C-H)	Olefins, Aromatic hydrocarbons
910-750	$\delta_{out\text{-}plane}$ (C-H)	Phenyl derivatives (including phenols)

Table 3 Auto-peaks in 2DCOS-IR synchronous spectra of Cabernet Sauvignon wines from three countries (65-110°C thermal perturbation)

Cabernet Sauvignon wines	Auto-peaks/cm^{-1} (threshold: 60% of relative intensity)					
Chile	**1648**	1516	**1164**	1044	**994**		
China	1721	1560	1512	1164	**1044**		
Australia	**1720**	**1626**	1448	1408	1164	**1042**	965

Peaks in bold are the strong auto-peaks.

Table 4 Auto-peaks in 2DCOS-IR synchronous spectra of Cabernet Sauvignon wines from different countries (110-120°C thermal perturbation)

Cabernet Sauvignon wines	Auto-peaks/cm^{-1} (threshold: 60% of relative intensity)							
Chile	**1580**	**1512**	1435	1250	**1152**	962	884	
China	**1650**	1408	1152	1042				
Australia	**1720**	**1580**	**1512**	1448	**1160**	**1030**	962	884

Peaks in bold are the strong auto-peaks.

Table 5 Parameters for geographical origin of SIMCA model

Parameters	Values
Score expansion	1.5
Residual expansion	1.0
Analysis range	4000-650 cm^{-1}
weight coefficient	Noise weighting, atmospheric weighting, Savitzky-Golay 5 point smoothing
Baseline correction	Resolution Weighting
Normalization	MSC

Table 6 Between-class distance PCs for three wines from different countries (Australia, Chile and China) of SIMCA model

Cabernet Sauvignon wines	Australia	Chile	China
Australia	-	2.1	2.13
Chile	2.1	-	2.25
China	2.13	2.25	-

Different samples were separated when the distance is >1.

Table 7 The preliminary assignment of main characteristic absorption peaks of FT-NIR spectra of Cabernet Sauvignon wines.

Peak Position（cm^{-1}）	Base Group and Vibration Mode	Main attribution
6845	*v* (O-H)	H_2O, Ethanol
5955-5866	C-H_3 stretch first overtone<br>C-H stretch first overtone	Aromatic compounds containing C-H, Ethanol
5610	C-H stretch first overtone and *v* (O-H)	Sucrose, Fructose and glucose
5226	O-H stretch overtone	H_2O, Ethanol
4411	C-H stretch combination<br>O-H stretch overtone	Alcohols, Carbohydrates, Aromatic compounds
4342	C-H stretch combination<br>O-H stretch overtone	Phenols, Organic carboxylic acids and Carbohydrates

Table 8 The Eigen analysis of spectrum contribution and analysis spectrum contribution for discriminating Cabernet Sauvignon wines from different countries

PC	Eigenvalues of full-wave bands	Eigenvalues of selected bands
1	61.5938	69.1762
2	81.5830	83.3695
3	88.0312	98.2097
4	93.1136	99.0502
5	94.6768	99.3153
6	95.4306	99.3953
7	95.9870	99.4692
8	96.3877	99.5237
9	96.7504	99.5660

Table 9 Validation results for wines from different countries (Australia, Chile and China) of SIMCA model

No.	Sample ID	Specified material	Identified material	Result	Specified material total distance ratio	Specified material distance ratio limit	Specified material model distance	Specified material residual distance
1	AC1-1	AC	AC	Passed	0.9617	1.0000	0.0000	1.1358
2	AC10-1	AC	AC	Passed	0.8593	1.0000	0.0000	1.0148
3	AC11-2	AC	AC	Passed	0.8484	1.0000	0.0000	1.0019
4	AC12-2	AC	AC	Passed	0.8004	1.0000	0.0000	0.9452
5	AC13-2	AC	AC	Passed	0.7747	1.0000	0.0000	0.9149
6	AC14-2	AC	AC	Passed	0.9232	1.0000	0.0000	1.0903
7	AC15-2	AC	AC	Passed	0.7614	1.0000	0.0000	0.8891
8	AC16-2	AC	AC	Passed	0.9665	1.0000	0.0000	1.1414
9	AC17-2	AC	AC	Passed	0.9580	1.0000	0.0000	1.1313
10	AC18-2	AC	AC	Passed	0.8448	1.0000	0.0000	0.9977
11	AC19-2	AC	AC	Passed	0.9292	1.0000	0.0000	1.0974
12	AC2-1	AC	AC	Passed	0.9702	1.0000	0.0000	1.1458
13	AC20-2	AC	AC	Passed	0.9382	1.0000	0.0000	1.1079
14	AC21-3	AC	AC	Passed	0.8561	1.0000	0.0000	1.0111
15	AC22-3	AC	AC	Passed	0.9080	1.0000	0.0000	1.0723
16	AC23-3	AC	AC	Passed	0.8071	1.0000	0.0000	0.9531
17	AC24-3	AC	AC	Passed	0.8652	1.0000	0.0000	1.0218
18	AC25-3	AC	AC	Passed	0.8918	1.0000	0.0000	1.0532
19	AC26-3	AC	AC	Passed	0.8148	1.0000	0.0000	0.9623
20	AC27-3	AC	AC	Passed	0.9062	1.0000	0.0000	1.0701
21	AC28-3	AC	AC	Passed	0.9145	1.0000	0.0000	1.0800
22	AC29-3	AC	AC	Passed	0.8721	1.0000	0.0000	1.0299
23	AC3-1	AC	AC	Passed	0.8396	1.0000	0.0000	0.9915
24	AC30-3	AC	AC	Passed	0.8943	1.0000	0.0000	1.0561
25	AC31-3	AC	AC	Passed	0.8603	1.0000	0.0000	1.0159
26	AC32-2	AC	AC	Passed	0.8684	1.0000	0.0000	1.0256
27	AC33-3	AC	AC	Passed	0.9383	1.0000	0.0000	1.1081
28	AC34-3	AC	AC	Passed	0.8534	1.0000	0.0000	1.0078
29	AC35-3	AC	AC	Passed	0.9052	1.0000	0.0000	1.0690
30	AC36-3	AC	Other	Failed	1.0523	1.0000	0.0000	1.2427
31	AC37-3	AC	Other	Failed	1.0529	1.0000	0.0000	1.2435
32	AC38-3	AC	AC	Passed	0.8608	1.0000	0.0000	1.0166
33	AC39-3	AC	AC	Passed	0.8478	1.0000	0.0000	1.0012
34	AC4-1	AC	AC	Passed	0.8676	1.0000	0.0000	1.0246
35	AC40-3	AC	AC	Passed	0.8478	1.0000	0.0000	1.0012
36	AC48-1	AC	AC	Passed	0.9278	1.0000	0.0000	1.0957
37	AC49-2	AC	AC	Passed	0.8400	1.0000	0.0000	0.9920
38	AC5-1	AC	AC	Passed	0.9907	1.0000	0.0000	1.1699

39	AC50-2	AC	AC	Passed	0.8112	1.0000	0.0000	0.9580
40	AC51-3	AC	AC	Passed	0.9975	1.0000	0.0000	1.1780
41	AC52-2	AC	AC	Passed	0.7446	1.0000	0.0000	0.8794
42	AC53-1	AC	AC	Passed	0.8313	1.0000	0.0000	0.9817
43	AC54-1	AC	AC	Passed	0.9880	1.0000	0.0000	1.1688
44	AC55-1	AC	Other	Failed	1.0044	1.0000	0.0000	1.1862
45	AC56-2	AC	AC	Passed	0.9059	1.0000	0.0000	1.0698
46	AC57-1	AC	AC	Passed	0.8547	1.0000	0.0000	1.0094
47	AC58-1	AC	AC	Passed	0.9887	1.0000	0.0000	1.1677
48	AC59-2	AC	AC	Passed	0.9396	1.0000	0.0000	1.1096
49	AC6-1	AC	AC	Passed	0.8035	1.0000	0.0000	0.9489
50	AC60-2	AC	Other	Failed	1.0706	1.0000	0.0000	1.2643
51	AC61-3	AC	AC	Passed	0.9761	1.0000	0.0000	1.1527
52	AC7-1	AC	AC	Passed	0.8386	1.0000	0.0000	0.9903
53	AC8-1	AC	AC	Passed	0.9091	1.0000	0.0000	1.0736
54	AC9-1	AC	Other	Failed	1.4881	1.0000	0.0000	1.7574
55	CLC1-1	CLC	Other	Failed	1.0050	1.0000	0.0000	1.1868
56	CLC10-1	CLC	CLC	Passed	0.9272	1.0000	0.0000	1.0949
57	CLC11-2	CLC	CLC	Passed	0.8915	1.0000	0.0000	1.0529
58	CLC12-2	CLC	CLC	Passed	0.8185	1.0000	0.0000	0.9666
59	CLC13-2	CLC	CLC	Passed	0.9312	1.0000	0.0000	1.0997
60	CLC14-2	CLC	CLC	Passed	0.8953	1.0000	0.0000	1.0573
61	CLC15-2	CLC	CLC	Passed	0.9674	1.0000	0.0000	1.1425
62	CLC16-2	CLC	CLC	Passed	0.8785	1.0000	0.0000	1.0375
63	CLC17-2	CLC	CLC	Passed	0.8513	1.0000	0.0000	1.0054
64	CLC18-2	CLC	CLC	Passed	0.9311	1.0000	0.0000	1.0996
65	CLC19-2	CLC	CLC	Passed	0.7939	1.0000	0.0000	0.9376
66	CLC2-1	CLC	CLC	Passed	0.9774	1.0000	0.0000	1.1543
67	CLC20-2	CLC	CLC	Passed	0.9113	1.0000	0.0000	1.0762
68	CLC21-2	CLC	CLC	Passed	0.9268	1.0000	0.0000	1.0945
69	CLC22-2	CLC	CLC	Passed	0.9848	1.0000	0.0000	1.1630
70	CLC23-2	CLC	Other	Failed	1.0471	1.0000	0.0000	1.2365
71	CLC24-2	CLC	CLC	Passed	0.7945	1.0000	0.0000	0.9383
72	CLC25-2	CLC	CLC	Passed	0.9782	1.0000	0.0000	1.1552
73	CLC26-2	CLC	CLC	Passed	0.9975	1.0000	0.0000	1.1780
74	CLC27-2	CLC	CLC	Passed	0.8783	1.0000	0.0000	1.0372
75	CLC28-2	CLC	CLC	Passed	0.9948	1.0000	0.0000	1.1748
76	CLC29-2	CLC	CLC	Passed	0.9181	1.0000	0.0000	1.0843
77	CLC3-1	CLC	CLC	Passed	0.9091	1.0000	0.0000	1.0736
78	CLC30-2	CLC	CLC	Passed	0.9404	1.0000	0.0000	1.1106
79	CLC31-3	CLC	CLC	Passed	0.8747	1.0000	0.0000	1.0329
80	CLC32-3	CLC	CLC	Passed	0.9092	1.0000	0.0000	1.0738

81	CLC33-3	CLC	CLC	Passed	0.9662	1.0000	0.0000	1.1410
82	CLC34-3	CLC	CLC	Passed	0.8416	1.0000	0.0000	0.9939
83	CLC35-3	CLC	CLC	Passed	0.9718	1.0000	0.0000	1.1476
84	CLC36-3	CLC	CLC	Passed	0.9087	1.0000	0.0000	1.0732
85	CLCL37-3	CLC	CLC	Passed	0.7876	1.0000	0.0000	0.9301
86	CLC38-3	CLC	CLC	Passed	0.9329	1.0000	0.0000	1.1017
87	CLC39-3	CLC	CLC	Passed	0.8176	1.0000	0.0000	0.9656
88	CLC4-1	CLC	CLC	Passed	0.9492	1.0000	0.0000	1.1210
89	CLC40-3	CLC	Other	Failed	1.0039	1.0000	0.0000	1.1856
90	CLC41-3	CLC	CLC	Passed	0.7697	1.0000	0.0000	0.9090
91	CLC42-3	CLC	CLC	Passed	0.8427	1.0000	0.0000	0.9952
92	CLC43-1	CLC	CLC	Passed	0.8102	1.0000	0.0000	0.9569
93	CLC44-1	CLC	CLC	Passed	0.7781	1.0000	0.0000	0.9189
94	CLC45-1	CLC	Other	Failed	1.0025	1.0000	0.0000	1.1840
95	CLC46-1	CLC	CLC	Passed	0.9768	1.0000	0.0000	1.1535
96	CLC47-1	CLC	CLC	Passed	0.9103	1.0000	0.0000	1.0750
97	CLC48-2	CLC	CLC	Passed	0.9309	1.0000	0.0000	1.0994
98	CLC49-2	CLC	CLC	Passed	0.8294	1.0000	0.0000	0.9794
99	CLC5-1	CLC	CLC	Passed	0.7595	1.0000	0.0000	0.8969
100	CLC50-2	CLC	Other	Failed	1.1078	1.0000	0.0000	1.3083
101	CLC51-2	CLC	CLC	Passed	0.8181	1.0000	0.0000	0.9661
102	CLC59-1	CLC	CLC	Passed	0.8733	1.0000	0.0000	1.0313
103	CLC6-1	CLC	CLC	Passed	0.8773	1.0000	0.0000	1.0361
104	CLC60-2	CLC	CLC	Passed	0.8260	1.0000	0.0000	0.9755
105	CLC61-3	CLC	Other	Failed	1.0159	1.0000	0.0000	1.1997
106	CLC62-3	CLC	CLC	Passed	0.9017	1.0000	0.0000	1.0649
107	CLC7-1	CLC	CLC	Passed	0.9264	1.0000	0.0000	1.0940
108	CLC8-1	CLC	CLC	Passed	0.9188	1.0000	0.0000	1.0851
109	CLC9-1	CLC	CLC	Passed	0.7992	1.0000	0.0000	0.9438
110	CNC1-1	CNC	CNC	Passed	1.2765	1.0000	0.0000	1.4892
111	CNC10-1	CNC	CNC	Passed	0.7971	1.0000	0.0000	0.9299
112	CNC11-2	CNC	Other	Failed	1.0738	1.0000	0.0000	1.2527
113	CNC12-2	CNC	CNC	Passed	0.9207	1.0000	0.0000	1.0741
114	CNC13-2	CNC	CNC	Passed	0.8390	1.0000	0.0000	0.9788
115	CNC15-2	CNC	CNC	Passed	0.8040	1.0000	0.0000	0.9379
116	CNC16-2	CNC	Other	Failed	1.1597	1.0000	0.0000	1.3529
117	CNC17-2	CNC	Other	Failed	1.1227	1.0000	0.0000	1.3097
118	CNC18-2	CNC	CNC	Passed	0.9127	1.0000	0.0000	1.0648
119	CNC19-2	CNC	CNC	Passed	0.9754	1.0000	0.0000	1.1379
120	CNC2-1	CNC	CNC	Passed	0.9077	1.0000	0.0000	1.0590
121	CNC20-2	CNC	CNC	Passed	0.7914	1.0000	0.0000	0.9232

122	CNC21-3	CNC	CNC	Passed	0.8238	1.0000	0.0000	0.9610
123	CNC22-3	CNC	CNC	Passed	0.8650	1.0000	0.0000	1.0091
124	CNC23-3	CNC	CNC	Passed	0.7930	1.0000	0.0000	0.9252
125	CNC24-3	CNC	CNC	Passed	0.8537	1.0000	0.0000	0.9960
126	CNC25-3	CNC	CNC	Passed	0.7899	1.0000	0.0000	0.9215
127	CNC26-3	CNC	Other	Failed	1.0892	1.0000	0.0000	1.2706
128	CNC27-3	CNC	CNC	Passed	0.9504	1.0000	0.0000	1.1088
129	CNC28-3	CNC	CNC	Passed	0.8332	1.0000	0.0000	0.9721
130	CNC29-3	CNC	Other	Failed	1.0665	1.0000	0.0000	1.2442
131	CNC3-1	CNC	Other	Failed	1.0495	1.0000	0.0000	1.2244
132	CNC30-3	CNC	CNC	Passed	0.9329	1.0000	0.0000	1.0883
133	CNC31-1	CNC	CNC	Passed	0.9830	1.0000	0.0000	1.1468
134	CNC32-1	CNC	CNC	Passed	0.9660	1.0000	0.0000	1.1270
135	CNC33-1	CNC	Other	Failed	1.1084	1.0000	0.0000	1.2931
136	CNC34-1	CNC	Other	Failed	1.1188	1.0000	0.0000	1.3052
137	CNC36-2	CNC	CNC	Passed	0.8604	1.0000	0.0000	1.0037
138	CNC37-2	CNC	CNC	Passed	0.8609	1.0000	0.0000	1.0043
139	CNC38-3	CNC	CNC	Passed	0.8566	1.0000	0.0000	0.9994
140	CNC39-3	CNC	CNC	Passed	0.8880	1.0000	0.0000	1.0360
141	CNC4-1	CNC	CNC	Passed	0.9184	1.0000	0.0000	1.0715
142	CNC40-2	CNC	CNC	Passed	0.7869	1.0000	0.0000	0.9180
143	CNC47-1	CNC	CNC	Passed	0.8331	1.0000	0.0000	0.9719
144	CNC48-2	CNC	CNC	Passed	0.9826	1.0000	0.0000	1.1463
145	CNC49-1	CNC	CNC	Passed	0.9833	1.0000	0.0000	1.1471
146	CNC5-1	CNC	Other	Failed	1.0903	1.0000	0.0000	1.2720
147	CNC50-1	CNC	CNC	Passed	0.8184	1.0000	0.0000	0.9548
148	CNC51-2	CNC	Other	Failed	1.0874	1.0000	0.0000	1.2685
149	CNC52-2	CNC	CNC	Passed	0.9167	1.0000	0.0000	1.0694
150	CNC53-2	CNC	CNC	Passed	0.8574	1.0000	0.0000	1.0002
151	CNC54-1	CNC	CNC	Passed	0.9277	1.0000	0.0000	1.0823
152	CNC55-1	CNC	Other	Failed	1.0754	1.0000	0.0000	1.2546
153	CNC56-2	CNC	Other	Failed	1.1045	1.0000	0.0000	1.2885
154	CNC57-3	CNC	CNC	Passed	0.8760	1.0000	0.0000	1.0220
155	CNC6-1	CNC	CNC	Passed	0.9325	1.0000	0.0000	1.0879
156	CNC8-1	CNC	CNC	Passed	0.9075	1.0000	0.0000	1.0587
157	CNC9-1	CNC	CNC	Passed	0.9370	1.0000	0.0000	1.0931

AC： Australian Cabernet Sauvignon; CLC: Chilean Cabernet Sauvignon; CNC: Chinese Cabernet Sauvignon
